# Advances in Hydrogel Adhesives for Gastrointestinal Wound Closure and Repair

**DOI:** 10.3390/gels9040282

**Published:** 2023-03-31

**Authors:** Xingyu Hu, Mark W. Grinstaff

**Affiliations:** Departments of Chemistry and Biomedical Engineering, Boston University, Boston, MA 02215, USA

**Keywords:** hydrogel, adhesive, gastrointestinal tract, wound healing, polymers, mechanical properties

## Abstract

Millions of individuals undergo gastrointestinal (GI) tract surgeries each year with common postoperative complications including bleeding, perforation, anastomotic leakage, and infection. Today, techniques such as suturing and stapling seal internal wounds, and electrocoagulation stops bleeding. These methods induce secondary damage to the tissue and can be technically difficult to perform depending on the wound site location. To overcome these challenges and to further advance wound closure, hydrogel adhesives are being investigated to specifically target GI tract wounds because of their atraumatic nature, fluid-tight sealing capability, favorable wound healing properties, and facile application. However, challenges remain that limit their use, such as weak underwater adhesive strength, slow gelation, and/or acidic degradation. In this review, we summarize recent advances in hydrogel adhesives to treat various GI tract wounds, with a focus on novel material designs and compositions to combat the environment-specific challenges of GI injury. We conclude with a discussion of potential opportunities from both research and clinical perspectives.

## 1. Introduction

The gastrointestinal tract (GI) contains all the major organs of the digestive system, including the esophagus, stomach, small intestine (duodenum, jejunum, and ileum), and large intestine (cecum, colon, and rectum) [1]. It is essential for the transportation, digestion, and absorption of food. Today, GI surgeries remove an inflamed or cancerous part of the GI tract as a consequence of cancer, gallbladder disease, or inflammatory bowel disease, or repair a perforation or anastomotic leak [2,3]. Traditional open surgeries occur through a large incision in the abdomen, typically used for complex conditions that require extensive dissection [4]. Minimally invasive surgeries, on the other hand, require smaller incisions and are less painful with faster recovery at reduced risk of infection, and include endoscopy, laparoscopy, and robotic surgery [4,5].

**Figure 1:**
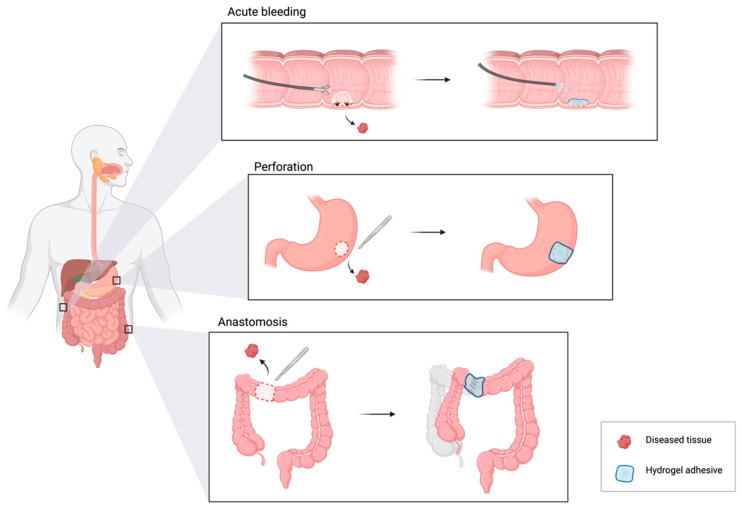
Hydrogel adhesives for different types of gastrointestinal (GI) wound closure and repair. Created with BioRender.com (accessed on 15 February 2023).

The surgical closure of GI wounds is key to restoring GI structure and function [6]. The management of GI wounds largely depends on the patient’s condition and the site’s size, location, and severity, ranging from acute bleeding to full-thickness perforation to anastomotic leaks (Figure 1) [7]. Sutures and staples are considered the “gold standard” for open surgery wound closure [8]. To better adapt to minimally invasive techniques, laparoscopic or endoscopic closures of GI wounds utilize metal clips, stents, or suturing devices for different sizes and locations of GI defects [9]. Despite the excitement surrounding the early evidence concerning minimally invasive closure techniques, there are many inherent disadvantages associated with suture-based tissue sealing that include: (1) secondary damage to tissue due to needle piercing; (2) complicated, time-consuming technical processes that require high surgical skills; (3) insufficient containment of fluid leaks; and (4) infection, inflammation, and delayed healing at the site [6,8,9,10,11]. Therefore, there is a substantial interest and need for the development of atraumatic, minimally invasive, and easy-to-apply GI wound closure technologies to provide fluid-tight sealing and promote wound healing for different types of GI defects.

Recently, hydrogel adhesives are emerging as an attractive alternative to sutures and staples for treating internal tissue wounds including wounds present in the GI tract. Hydrogels are three-dimensional, hydrophilic, crosslinked polymer networks that absorb and retain large amounts of water to maintain a gel-like swollen state [12]. The hydrogel compositions are diverse and span natural polymers, such as polysaccharides (chitosan, cellulose, alginate), polyamides (collagen), and other biological polymers, as well as synthetic polymers such as polycaprolactone (PCL), poly(ethylene glycol) (PEG), poly(vinyl alcohol) (PVA), and poly(lactic acid) (PLA), or mixtures [12]. Chemical crosslinking through covalent bonding, physical crosslinking through molecular entanglement, hydrogen bonding, hydrophobic association, or complexation via polyelectrolyte interactions afford the hydrogel state [12]. To form robust adhesion with the tissue under physiological conditions, various adhesion mechanisms are being investigated (as discussed later), including physical interactions (hydrogen bonds, van der Waals forces, electrostatic interactions, hydrophobic interactions, mechanical interlocking, etc.), chemical crosslinking (*N*-hydroxy succinimide (NHS)-ester-amine coupling, Schiff-base reaction, Michael-type addition, free radical polymerization, etc.), and bionic adhesion (mussel-inspired, gecko-inspired, and barnacle-inspired adsorption, etc.) [6,8,13,14]. Notably, strong and stable wet adhesion is achieved by combining different hydrogel backbones, preparation techniques, adhesion topologies, and bond chemistries [14]. The resulting swelling behavior, biodegradability, and mechanical strength of the hydrogel depend on the molecular weight of the polymer, the type and degree of crosslinking, and the secondary interactions between the polymer chains [6,12,15]. Normally, hydrogels in fully swollen states are viscoelastic, low in interfacial angle with biological fluids, and structurally similar to natural living tissues, contributing to their favorable biocompatibility [12]. Due to their hydrophilicity, biodegradability, and biocompatibility, hydrogels also play a prime role in other biomedical applications, including drug delivery, tissue engineering, and medical implants [12,16].

Specific to the application of wound closure, hydrogel adhesives are advantageous over sutures and staples for treating internal tissue wounds due to their unique advantages which include: (1) large swelling capacity and high water content, creating a moist environment while absorbing exudate; (2) full coverage of wound area, achieving fluid-tight sealing of the tissue defect; (3) tunable mechanical properties that can match the underlying tissue, distributing the stress concentration at the interface; (4) porous and bioactive structure that bears similarity to native extracellular matrix (ECM), providing a favorable microenvironment for wound healing; (5) a carrier for cells, drugs, and biological factors; (6) natural mechanical barrier to protect the wound from infection; (7) biodegradation without the need for removal; (8) suitable for different types and shapes of wound; (9) ease of delivery and application; (10) good biocompatibility; and (11) highly tailorable for a variety of tissue conditions [6,8,11,17]. With an increasing understanding of the underlying pathophysiology of tissues and the healing impairment induced by surgical procedures, there is a push for hydrogel adhesives designed for a given wound condition [11,18].

The application of hydrogel adhesives in GI wound repair poses significant technical and scientific challenges. The acidic environment of the GI tract, characterized by pH levels ranging from 1.0–2.5 in the stomach to 6.5–7.5 in the intestine, as well as exposure to corrosive digestive fluids containing digestive enzymes, bacteria, and bile, can result in degradation of crosslinked hydrogel networks [19,20]. Additionally, the heavy gel-mucous layer covering the surface of the GI tract, composed of large, highly glycosylated proteins, presents challenges for successful wet adhesion [21]. The dynamic movements of the GI tract, including peristalsis and segmentation associated with processes of digestion, further require the hydrogel adhesives to exhibit strong mechanical properties such as toughness, ductility, and fatigue resistance [20,22]. These challenges also offer opportunities for scientific investigation and engineering design to create hydrogel adhesives that provide effective and long-lasting wound healing within the GI tract. Furthermore, the route of administration, whether through an endoscope, laparoscope, or during open surgery, and the condition of the wound, whether it is loss of mucosa or submucosa, full-thickness wall perforation, or failure of an anastomosis, dictate distinct design requirements for hydrogel adhesives. 

**Figure 2:**
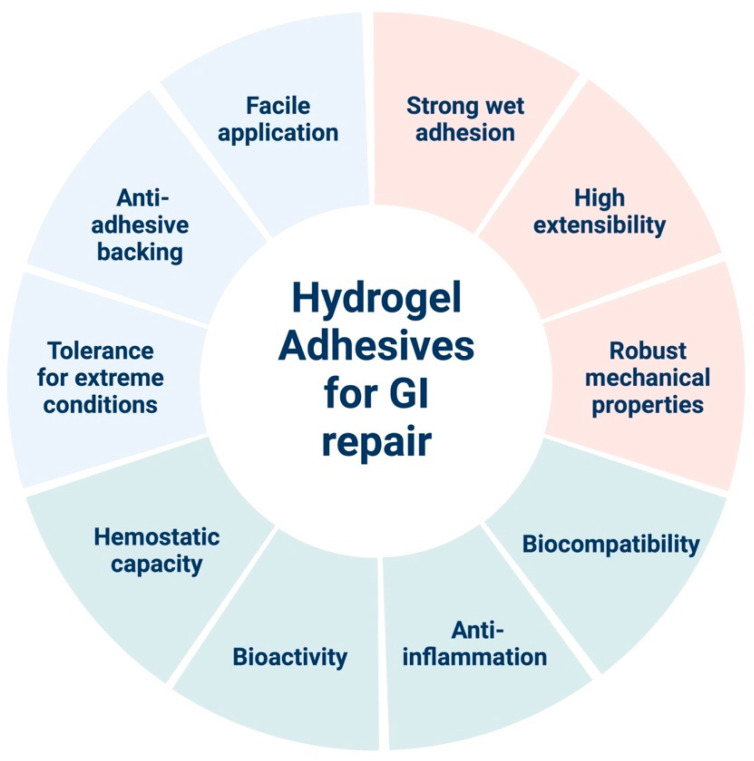
General principles for the design of hydrogel adhesives for GI wound closure and repair. Created with BioRender.com (accessed on 15 February 2023).

In this review, we focus on three GI wound conditions: acute bleeding, perforation, and anastomotic leak. First, we describe the disease condition and current standards and propose a list of design requirements for an ideal hydrogel adhesive (Figure 2). Then, we present recent advances in hydrogel adhesive designs and compositions to target the specific type of wound, underlining the mechanisms for crosslinking and wet adhesion, the strategies to promote wound healing, and the efficacies of the hydrogel in vivo. At the end of each section, we provide a summary to highlight common themes and provide directions for future improvements.

## 2. Acute GI Bleeding

### 2.1. Disease Condition and Current Standards

GI bleeding is a major cause of morbidity and mortality worldwide resulting in a hospitalization rate of 21 per 100,000 adults, and a mortality rate of 2% to 15% [23]. There are various causes of GI bleeding, including hemorrhoids, peptic ulcers, tears, inflammation, colonic polyps, or cancer in the colon, stomach, or esophagus [24]. Endoscopic intervention is the gold standard for GI hemostasis and wound healing. Endoscopic mucosal resection (EMR) and endoscopic submucosal dissection (ESD) are well-established interventions to remove early and middle-stage tumors from the gastrointestinal tract and their use reduces the incidence and mortality of GI cancer [25]. However, adverse events arise from endoscopic resection including intraprocedural bleeding, delayed bleeding, delayed perforation, hemorrhaging, and sepsis [25,26,27], with delayed bleeding being the most common and affecting 3–12% of patients [28]. Clinically, electrocoagulation, argon plasma coagulation, and mechanical clip placement are used to control bleeding and close the mucosal defect post EMR or ESD [29]. Although these techniques are effective in achieving acute hemostasis in about 90% of cases [30], there is limited evidence supporting their efficacy in preventing delayed bleeding and delayed perforation [26]. Additionally, electrocoagulation carries the risk of thermal injury, perfusion, and post-polypectomy coagulation syndrome [27]. Complete clip closure of the wound can be technically challenging depending on the bleeding site and endoscopist experience [31], or even impossible in 40% of cases due to the large wound size or poor accessibility [25].

In the last decade, several topical hemostats have been developed for surgical use, including formulations based on oxidized cellulose, gelatin, collagen, fibrin and thrombin, hyaluronic acid, and cyanoacrylates. All of these hemostats possess unique strengths, but none are ideal due to their weak mechanical properties, cytotoxicity, long procedural time, quick degradation, and/or risk of pathogen infection [30,32]. Recently, topical hemostatic powders such as Hemospray (TC-325), EndoClot, and Ankaferd Blood Stopper have been used to control bleeding [33]. The immediate control rate is in the range of 88–100%, with a rebleeding rate of 3–13% [33]. These powders do not precisely cover the target legion and lack bio-adhesion to the GI mucosa, thereby giving rise to the risk of re-bleeding. Additionally, existing powders are opaque, which can block the surgeon’s view of the operative field and hinder the surgical procedure [31].

### 2.2. Hydrogel Requirements

An ideal device for GI hemostasis requires: (1) rapid and long-term hemostatic effects; (2) easy use with minimal endoscopist skills; (3) sufficient adhesion and mechanical strength in a wet tissue environment; (4) anti-bacterial and anti-infection properties; and (5) biocompatibility. Hydrogel-based GI hemostats adhere and provide a mechanical barrier to promote rapid hemostasis. Due to the nature of endoscopic intervention, one prefers sprayable and injectable in situ forming hydrogels for ease of operation. Fast gelation of the hydrogel (gelation time < 5 min) is the number one design criterion for rapid hemostasis. Additionally, the hydrogel should withstand the burst pressure of arterial bleeding (burst pressure > 120 mmHg) and the mechanical motility of the GI tract with strong and stable wet adhesion capabilities that surpass that of the conventional surgical glues and match the GI epithelial turnover rate (adhesive strength > 10 kPa on wet tissue for more than 48 h). These requirements compel a multi-functional design of the hydrogel network and its adhesion mechanisms. 

### 2.3. Crosslinking and Adhesion Mechanisms

**Figure 3:**
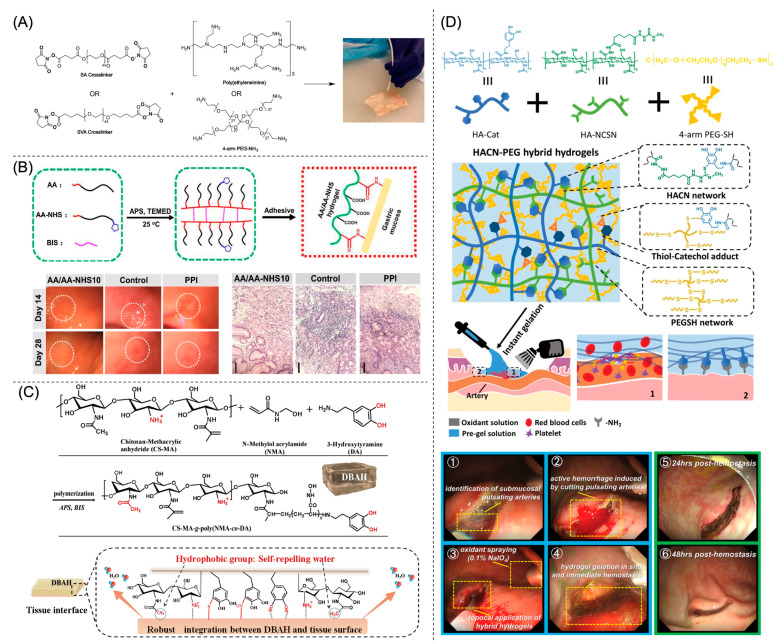
Hydrogel adhesives designed to treat acute GI bleeding. (**A**) A PEG-based injectable hydrogel formulation for colon polypectomies, reprinted with permission from Ref. [27]. Copyright 2021 American Chemical Society. (**B**) Hydrogels prepared by the free radical polymerization of AA and AA-NHS crosslinked by methylene bisacrylamide (BIS) stop acute bleeding and assist in wound healing, reprinted with permission from Ref. [42]. Copyright 2021 Springer Nature. (**C**) A dual adhesive hydrogel based on chitosan grafted with methacrylate (CS-MA), dopamine, and *N*-hydroxymethyl acrylamide shows strong bioinspired wet adhesion owing to the self-repelling water function of CS-MA, reprinted with permission from Ref. [43]. Copyright 2020 Elsevier. (**D**) A hybrid hydrogel comprised of dual networks of HA and PEG stabilized by NCSN-Cat coupling and disulfide bonds demonstrates rapid gelation, immediate hemostatic effect, and continued adhesion to the GI wall for more than 48 h in a pig acute hemorrhage model, reprinted with permission from Ref. [30]. Copyright 2021 Wiley-VCH GmbH.

Most hydrogels exhibit weak mechanical properties compared to GI tissues which possess a storage modulus (G′) ranging from 100 Pa to 10 kPa, with the proximal colon being the strongest [34]. PEG-based hydrogels containing crosslinked symmetrical tetra-arm macromonomers, of the same size, form homogenous structures with substantial mechanical strength comparable to natural cartilage [35]. Therefore, many studies report the use of multi-arm PEG as the base material due to its biocompatibility, ease of manufacturing, and high mechanical strength (Figure 3A) [27,36,37,38,39]. For example, a tetra-arm PEG-amine (NH_2_) gels with a NHS functionalized PEG (NHS-PEG-NHS) in 100 s [27]; a tetra-arm PEG with end-capped thiols (SH) gels with a tetra-arm PEG-maleimide through a thiol-ene reaction in 4 min [36]; a tetra-arm PEG-NH_2_ and a tetra-arm PEG-aldehyde (CHO) gel through Schiff base reactions in 25 s [37]. All three hydrogel formulations exhibit high storage moduli of over 5 kPa, good adhesive strengths of around 10 kPa, comparable to commercially available adhesives, and short in situ gelation time within 5 min, enabling rapid hemostasis [40]. However, their crosslinking mechanisms may not be optimal for the gastric environment (pH = 1.0–2.5). At this low pH, the amino groups will protonate and be inactive, and the Schiff base quickly hydrolyzes [37], leading to the fast disintegration of the hydrogel. In contrast, the pH ranges from 6.6–7.5 in the lower GI organs including the small intestine and colon [41]. Therefore, these reaction chemistries and formulations are appropriate for lower GI hemostasis and wound healing. However, a study using tetra-arm PEG hydrogels in a rat gastric wound model shows that only 16% of hydrogel remains at 48 h after application, which is deemed inadequate [36]. The weak adhesion mechanism, which relies on hydrogen bonding between the polymer and the hydrophilic groups on the tissue, is likely the cause. GI motility and peristalsis as well as epithelial cell turnover create a challenging environment for hydrogel adhesion.

Anchoring chemical adhesion moieties such as NHS and CHO that react with tissue amines will enhance adhesive strength. Adopting other crosslinking mechanisms may also improve the integrity and acidic stability of the hydrogel. Hydrogels prepared by the free-radical polymerization of acryloyl-6-aminocaproic acid (AA) and AA-NHS crosslinked by methylene bisacrylamide (BIS) exhibit comparable storage modulus and adhesive strength as the PEG gels. Notably, they also show good injectability, autonomous self-healing capacity, and stable adhesive behavior in gastric conditions, which facilitates good hemostatic performance by stopping acute bleeding and preventing delayed bleeding in a swine gastric hemorrhage model (Figure 3B) [42]. The hydrogels accelerate gastric wound healing by controlling inflammation, suppressing fibrosis, and promoting ECM remodeling and angiogenesis [42]. However, the complete gelation procedure requires 9 min, with an initial 3-min pre-polymerization step outside the body, adding complexity to clinical procedures and thus limiting its translational potential [42].

### 2.4. Enhancing Wet Adhesion

To enhance wet adhesion and hydrogel integrity in the GI environment, researchers install additional functional groups within hydrogels, including those inspired by nature. Mussels adhere strongly to diverse wet substrates via excreted proteins. The key component of mussel adhesion protein is 3,4-dihydroxyphenylalanine (L-DOPA). The mechanism of adhesion encompasses both physical interactions, such as hydrogen bonding, metal chelation, and π–π bonding, as well as chemical interactions, such as oxidation of polyphenols, Schiff base, or Michael addition reactions with the wet tissue surface [8]. A variety of polyphenols, including dopamine, tannic acid, and catechol (Cat), are under investigation to enhance wet adhesion. Xia et al. report a hybrid hydrogel comprised of hyaluronic acid (HA) modified with catechol (Cat) or thiourea (NCSN) and tetra-arm PEG with end-capped thiols (SH) for the treatment of acute upper GI hemorrhage (Figure 3D) [30]. The dual intertwined networks of HA and PEG endow the hydrogel with a G′ of over 8 kPa and shortens the in situ gelation time to less than 5 s after oxidation is induced for catechol-thiourea coupling and disulfide bond crosslinking. Due to the anchorage of Cat moieties on the tissue, the adhesion strength reaches 14 kPa and the burst pressure is 140 mmHg, higher than the normal maximum arterial pressure of 120 mmHg. Complete hemostasis occurs within 2 min of application and the hydrogel remains adherent for more than 48 h in a pig hemorrhage model, demonstrating rapid and long-term hemostatic effects of the hydrogel [30].

Another barrier to wet adhesion and sealing hemostasis is interfacial water, which is present as a film on tissue surfaces. To address this challenge, researchers incorporate hydrophobic components into the hydrogel to displace the interfacial water layer to facilitate subsequent chemical and physical bonding with the tissue [28,44,45,46]. Han et al. describe a novel dual adhesive hydrogel formed via an ammonium persulfate (APS) radical polymerization of chitosan grafted with methacrylate (CS-MA), dopamine, and *N*-hydroxymethyl acrylamide (Figure 3C). When in contact with water, the abundant hydrophobic residues of CS aggregate to repel interfacial water and the catechol and amine groups chemically and physically bond with the tissue surface. The hydrogel achieves an adhesive strength of over 34 kPa and a burst pressure up to 168 mmHg, and facilitates the hemostasis of a rabbit’s heart in vivo [43]. This formulation has not been evaluated for preventing GI bleeding. However, due to the cytotoxicity of the catalysts and the viscosity of the hydrogel solution, this hydrogel is pre-made and administered as a patch. Alternatively, microparticle-based injectable wound dressings with wet-adhesion stability are being developed based on hydrophobically-modified Alaska pollock gelatin via thermal crosslinking for GI hemostasis and wound healing [44]. Hydrophobic modification drastically enhances the mechanical properties and the underwater stability of the microparticles to up to 4 days. The microparticles also suppress fibrosis and inflammation in a rat skin wound healing model. Additionally, self-assembling peptide (SAP) hydrogels spontaneously form nanofibers and interact with the tissue surface through both hydrophobic and hydrophilic interactions [25,31,36,47,48]. The SAP hydrogel, also known as RADA16, consists of a fully synthetic 16-amino-acid polypeptide with a repeating sequence of R (positively charged arginine), A (hydrophobic alanine), and D (negatively charged aspartic acid). The monomer building blocks form crosslinked β-sheet structures via non-covalent interactions and mimic a natural extracellular matrix scaffold that adheres to the tissue [47]. SAP hydrogels are effective in controlling bleeding after EMR for gastric cancer, with an average time-to-hemostasis of 105 s [39,48].

### 2.5. Antibacterial Properties and Wound Healing

In addition to hemorrhage control and wet adhesion, bacteria mitigation and infection prevention are also crucial to an advantageous wound healing outcome. Exposure of GI wound tissues to pathogenic bacteria, such as *Pseudomonas aeruginosa*, results in enhanced collagen-degradation [6]. Traditional protein-based sealants such as fibrin glue and collagen patches are particularly susceptible to bacterial degradation—limiting their utility [6]. One approach to inhibit bacteria migration to the wound site is to create hydrogels with pore sizes smaller than the bacteria size and thus restrict bacterial mobility and migration to the tissue [27]. Alternatively, antibiotics such as tetracycline and vancomycin loaded into hydrogels exert antibacterial activity against gram-negative and gram-positive bacteria, respectively [37,49]. Chitosan is a nontoxic natural antimicrobial polymer and kills microbes by destabilizing the negatively charged membrane of the bacteria [50]. Hydrogels consisting of modified chitosan show anti-bacterial and anti-infection capabilities against *Escherichia coli* (*E. coli*) and *Staphylococcus aureus* (*S. aureus*), respectively, while maintaining biocompatibility [38,43].

### 2.6. Summary

Several hydrogel designs show promise to treat acute GI bleeding and facilitate wound healing. Due to the nature of endoscopic intervention, the design of hydrogel adhesives for GI hemostasis focuses on injectability for easy delivery and quick gelation for rapid hemostasis. However, a challenge remains to identify a hydrogel that balances facile application with prolonged hemostatic and adhesive capabilities without over-complicating the formulation. For example, strong wet adhesion may require longer gelation time and patch delivery [42,43]. On the other hand, forming more acid-tolerant hydrogel networks may entail the risk of cytotoxicity from required catalysts during in situ polymerization [42]. Excitingly, many of the formulations are still in their very early stages and significant research opportunities are present. Further design explorations and in vivo animal studies are needed to identify reliable hydrogel candidates for different location-specific GI hemorrhages followed by the first in-human trials to advance these materials to the clinic—as none currently exist.

## 3. GI Perforation

### 3.1. Disease Condition and Current Standards

Perforation is a hole that develops through the wall of a body organ. A gastric perforation (GP) is a full-thickness injury of the stomach wall with spillage of the gastric contents into the general peritoneal cavity [51]. Severe complications are often associated with GP including bleeding, sepsis, chemical peritonitis, bowel infarction, wound infection, and multi-organ failure [52,53,54]. The most common cause of gastric perforation is peptic ulcer disease, a chronic disease that results from an imbalance between endogenous protective factors of the gastric mucosa and aggressive factors, with a lifetime prevalence of 5–10% in the general population [55]. GP also arises from trauma, malignancy, intrinsic gastric pathology, or endoscopy-related interventional procedures including EMR and ESD [51]. An intestinal perforation (IP) is a loss of continuity of the bowel wall, resulting from a variety of disease processes such as ischemia, infection, erosion, and physical disruption, and causing complications such as sepsis, peritonitis, and anastomotic leakage [56].

Non-surgical management of perforation usually involves analgesia, intravenous antibiotics, and proton pump inhibitory medications (PPIs), but faces the risk of higher mortality rate if it fails [55]. Therefore, current treatment primarily relies on endoscopic closure and laparotomy for perforations less than 10 mm and open surgery for larger perforations [55,57]. Direct closure of the perforation with interrupted sutures and an omental pedicle plug is the most commonly used technique. However, this procedure results in a 7% suture leak rate with laparoscopic repair and is usually associated with severe tissue damage and inflammation caused by deep piercing and ischemia [54,55]. As a result, the estimated postoperative mortality rate for perforation ranges from 1.3% to 20%, with the 90-day mortality rate reaching up to 30% [55]. Therefore, there is an imperative need for the development of new biomaterials and techniques to improve perforation sealing and facilitate sutureless GI repair.

### 3.2. Hydrogel Requirements

To form fluid-tight sealing of GI perforation, an ideal hydrogel adhesive requires: (1) extremely strong and stable long-term wet adhesion; (2) mechanically robust network; (3) tolerance for extreme pH conditions (especially for gastric perforation); (4) excellent interfacial toughness, ductility, and fatigue resistance; (5) ease of application and use; and (6) biocompatibility. Unlike the hydrogels designed for GI hemostasis and wound healing, hydrogels here focus more on enhancing the mechanical properties (G′ > 6 kPa, interfacial toughness > 50 J/m^2^, burst pressure > 120 mmHg) and strengthening wet adhesion (adhesive strength > 10 kPa for over 7 days), as they need to sustain fluid pressure and prevent leakage before the perforation completely heals. Strong and durable interfacial bonds need to be established between hydrogels and the tissue surface to prevent adhesive failure, whereas robust, tough, and acid-tolerating networks need to be constructed for the bulk hydrogel to prevent cohesive failure. Additionally, the adhesive may be delivered endoscopically or laparoscopically as a glue or through open surgery as a patch depending on the location and size of the perforation.

### 3.3. Crosslinking and Adhesion Mechanisms

Hydrogen bonds can endow materials with high toughness, elasticity, and self-healing properties owing to their dynamic nature. By incorporating substantial amounts of free hydrogen bonding groups, such as carboxylic acid, amine, and alcohol, into the network, strong adhesion interfaces form between the hydrogel and the tissue surface. A hydrogel adhesive prepared by free radical polymerization of a bi-carboxyl-containing vinyl monomer *N*-acryloyl aspartic acid (AASP) shows good toughness, robust elasticity, fatigue resistance, strong adhesion to various tissues (120 kPa), and stability in simulated gastric fluid for more than 7 days [58]. The rich carboxyl groups on the side chains ensure both the formation of the hydrogel network and the interaction with the polar groups on the tissue interface [58]. Physically crosslinked polyelectrolytes also form strong hydrogen bonding interactions by diffusing into the tissue. A self-gelling and adhesive polyethyleneimine (PEI) and polyacrylic acid (PAA) powder effectively seals gastric perforation within 2 s in a rat model [59]. Due to the water-induced gelation mechanism, the PEI/PAA powder absorbs interfacial water to enhance wet adhesion and can be delivered endoscopically to fit irregularly shaped target sites. However, the hydrogel does not survive extreme pH environments (pH < 2 or pH > 11) because of its high charge density, limiting its application to nondigestive periods and only the lower GI tract [59].

**Figure 4:**
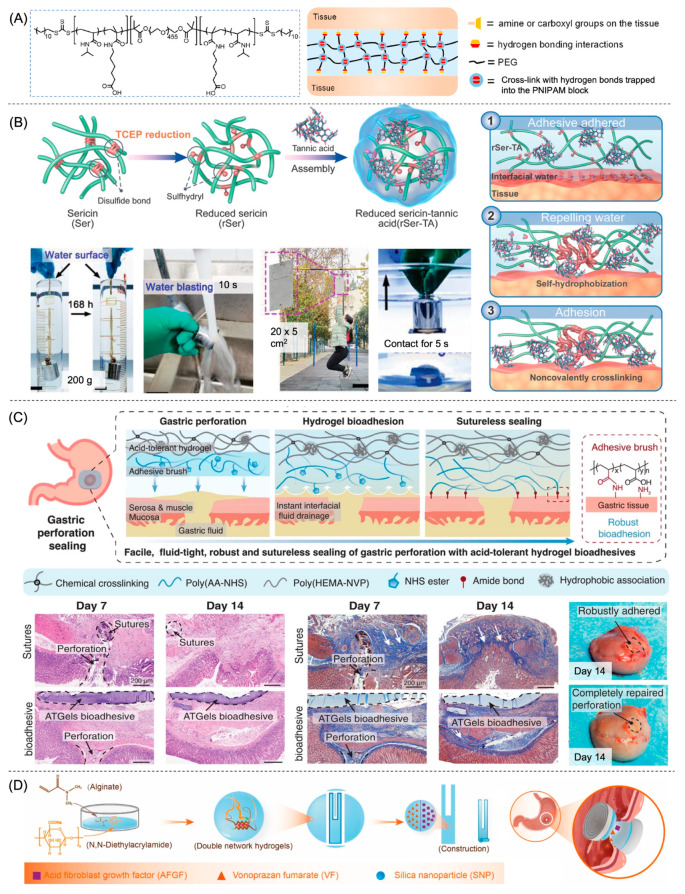
Hydrogel adhesives designed to treat GI perforation. (**A**) A hydrogel based on gastric environment-adaptive supramolecular assembly shows thermo-sensitivity, injectability, printability, and rapid self-healing capability, reprinted with permission from Ref. [39]. Copyright 2021 American Chemical Society. (**B**) A self-hydrophobized adhesive consisting of reduced silk sericin protein and tannic acid achieves extremely rapid, robust, and durable underwater adhesion, making it suitable for fluid leakage sealing and in vivo wound healing, reprinted with permission from Ref. [45]. Copyright 2022 Wiley-VCH GmbH. (**C**) An acid-tolerant hydrogel bioadhesive based on a hydrophobically crosslinked substrate and a dry adhesive polymer brush forms instant, atraumatic, fluid-tight, and sutureless sealing of gastric perforation and accelerates repair by alleviating inflammation and enhancing angiogenesis, reprinted with permission from Ref. [53]. Copyright 2022 Wiley-VCH GmbH. (**D**) A self-expandable, endoscopically deliverable, and hyperboloid-shaped hydrogel blocks gastric perforation and delivers vonoprazan fumarate (VF) and acidic fibroblast growth factor (AFGF) to regulate intragastric pH and promote wound healing, reprinted with permission from Ref. [52]. Copyright 2023 American Chemical Society.

Additionally, hydrogels based on dynamic covalent bonds are attractive due to their inherent self-healing ability and high cohesion on account of the strong covalent bonds [14]. A hydrogel patch adhesive, prepared through Schiff base reaction between tetra-PEG-CHO and carboxymethyl chitosan (CMCS), displays high storage modulus (25 kPa), high burst pressure (217.5 mmHg), and rapid self-healing under physiological conditions [38]. Wang et al. report an injectable hydrogel assembled from an ABA triblock copolymer composed of a middle PEG block and terminal temperature-responsive poly(*N*-isopropylacrylamide-co-*N*-acryloyl-6-aminocaproic acid) P(NIPAM-co-NA6ACA) blocks (Figure 4A) [39]. Upon application, the polymer solution transforms into a physical supramolecular hydrogel due to the hydrophobic interactions between the collapsed P(NIPAM-co-NA6ACA) blocks. The hydrogel self-heals in the acidic environment due to the synergy of hydrogen bonding and hydrophobic interactions, bestowing an added advantage. Its adhesive strength to porcine stomach, however, is relatively weak (6 kPa) owing to its mere hydrogen bonding adhesive mechanism [39].

### 3.4. Enhancing Wet Adhesion

As discussed above, biomimetic catechol-based mussel-inspired hydrogels are extensively studied for wet adhesion. However, it is a challenge to fabricate catechol-based hydrogels for perforation sealing because strong adhesion and cohesion of the hydrogel require drastically different pH conditions, respectively [60,61]. Alternatively, tannic acid (TA) is a natural plant-based polyphenol containing a high density of pyrogallol and catechol groups that functions as a physical crosslinker as well as an adhesive moiety to facilitate both cohesion and adhesion in acidic conditions [61,62]. Its dendritic structure provides multiple bonding sites for hydrogen bonding, ionic bonding, and hydrophobic interactions [61]. Additionally, TA adhesion increases when exposed to oxidants [60]. A gelatin methacrylate (GelMA) containing TA exhibits high stiffness (4.6 MPa), high adhesive strength (80 kPa), large deformability (277% elongation), and self-healing property [62]. Its stability in acidic conditions, however, is questionable due to its hydrolyzable ester linkages in GelMA. Acid-tolerant hydrogel adhesives based on dopamine-modified poly(γ-glutamic acid) (γ-PGA) and TA also exhibit high storage modulus (5 kPa), high adhesive strength (50 kPa), and large deformability (800% elongation). Notably, due to its abundant phenolic hydroxyl groups and complete physical crosslinking mechanism, the hydrogels display stable and robust adhesion in simulated gastric juice (pH = 1) for 7 days, significantly surpassing fibrin glue in acid-tolerating capacities [60].

To further enhance wet adhesion, interfacial water dehydration is necessary to remove the gap between the adhesive and the tissue [63]. There exist two distinct strategies: (1) increasing polymer hydrophobicity to break the hydration layer and displace the water at the molecular level; and (2) utilizing polymer hydrophilicity to absorb interfacial water at the microscopic level [63]. Glue-type injectable hydrogels usually adopt the first strategy to enhance molecular-level interfacial bonding as the polymers diffuse across the interfacial water and form mechanical interlocking with the tissue layer. Hydrophobically modified Alaska pollock gelatin microparticle-based wound dressings are of interest for treating not only acute GI bleeding but also ESD-induced perforation [28,44]. Gradual fusion of the microparticles occurs during the first 60 min of application via hydrophobic interactions to increase the burst strength of the hydrogel and improve its underwater stability on duodenum tissue ex vivo [28]. In another study, silica nanoparticles coat hydrogels to enhance the adsorption and entanglement between the hydrogel and the tissue surface [52]. The wet adhesive capability of biomimetic TA-based hydrogels is further strengthened by the inclusion of hydrophobic moieties. A self-hydrophobized adhesive co-assembled from disulfide-bond-hydrolyzed silk sericin protein and TA achieves extremely robust (>100 kPa for tissues) and durable (>7 days) underwater adhesion and seals a mouse small intestine perforation instantly (Figure 4B) [45]. The dissociation of disulfide bonds in silk sericin protein induces hydrophobic amino acid eversion, thereby leading to the self-aggregation of hydrophobic chains once exposed to water, repelling interfacial water, and enhancing subsequent interfacial physical crosslinking effects [45]. 

Patch-type hydrogels, on the other hand, frequently adopt the second strategy, also known as the dry-crosslinking mechanism, to remove interfacial water and facilitate subsequent crosslinking. Upon contact with wet surfaces, a dry double-sided tape made from the combination of a biopolymer (gelatin or chitosan) and crosslinked PAA grafted with NHS ester instantly dries the interfacial water and swells [40]. Temporary adhesion via hydrogen bonding occurs within the first 5 s, followed by covalent adhesion between the NHS ester groups and the primary amine groups on the tissue surface, enabling its potential application to seal air-tight lung lobes as well as fluid-tight perforated stomachs and small intestines [40]. Additionally, the degradation and mechanical properties of the network are controlled by tuning the composition of the biopolymer component [40]. To integrate both strategies of wet adhesion, Liu et al. describe a half-dry adhesive for rapid gastric perforation and traumatic pneumothorax sealing [57]. The hydrogel first repels the majority of interfacial liquid based on its moderate hydrophilicity, then absorbs trapped residues and vicinal tissue fluid to enhance topological adhesion, and finally bonds to the tissue surface through physical interactions [57]. With the combined strategy, the PAA-silk fibroin (SF) based adhesive achieves excellent adhesion energy (600 J/m^2^) and burst pressure (1500 mmHg) for more than 24 h.

### 3.5. Multifunctionality through Interpenetrating Polymer Networks

Interpenetrating polymer network (IPN) hydrogels are polymer composites composed of two or more crosslinked networks that are topologically entangled and cannot be separated without disrupting existing chemical bonds [64]. Compared to single network hydrogels, IPNs possess more widely controllable physical properties and more versatile functionalities [65]. Due to the multitude of design requirements needed for hydrogels to target GI perforation, various IPN hydrogels are being developed to improve on at least one property of the single network, such as mechanical reinforcement, adhesion enhancement, or better energy dissipation. These IPN hydrogels typically comprise a covalently crosslinked rigid network and a physically crosslinked soft network. The covalent network acts to reinforce the mechanical properties, stabilize the polymer network, and prevent dissociation in extreme pH environments [40,66]. The physical network allows for energy dissipation, good ductility and flexibility, and self-healing properties [54].

Sodium alginate (SA) physically crosslinked with calcium ions (Ca^2+^) serves as a second network in a poly(acrylamide) (PAM)-based covalent network to enhance energy dissipation in multiple instances [52,67]. One of the challenges for GI perforation repair lies in the excessive swelling of hydrogels, which may cause a mismatch strain between the hydrogel and the tissue, thereby reducing the overall adhesion performance [67]. To address this issue, nano-hydroxyapatites are embedded into the PAM-based network to function as ionic nano-reservoirs and gradually release Ca^2+^ in acidic environments, building a second SA-based network to inhibit swelling of the hydrogel in gastric juice [67]. Silk fibroin (SF) is a natural multi-domain protein that is also being incorporated into IPN hydrogel adhesives owing to its superior strength and stretchability [68]. Liu et al. report a half-dry adhesive for wet adhesion consisting of a SF semi-interpenetrating network and a PAA covalent network [57]. After the addition of acrylic acid, the heavy chains of SF can rearrange from *α*-helical conformations to antipolar-antiparallel beta-sheets (*β*-sheets), providing additional physical crosslinks and enhancing the bulk toughness of the hydrogel [57]. Dynamic covalent bonding networks also complement covalent networks to enhance mechanical strength and provide energy dissipation due to their thermodynamically controlled reversible junctions [14]. Chen et al. report an injectable hydrogel consisting of a bioactive, transglutaminase (TG)-crosslinked gelatin network and a dynamic, borate-crosslinked poly-*N*-[tris(hydroxymethyl)methyl]acrylamide (PTH) network [54]. The primary gelatin network covalently crosslinks with tissue amines via TG to provide adhesion whereas the secondary PTH network favors energy dissipation through its reversible boronic-ester bonds [54].

Due to the versatility and flexible functionalities of IPN hydrogels, researchers are adopting various strategies to construct IPN hydrogels with not only enhanced mechanical properties but also acid-tolerating capabilities for GI perforation applications. Bian et al. describe a fit-to-shape sealant enhanced by photo-initiated crosslinking to treat wounds inside the stomach with the existence of gastric acid [66]. The primary network of the hydrogel is based on dynamic Schiff-base linkages between chitosan and benzaldehyde-terminated PEG to endow the hydrogel with shear-thinning injectable properties. The secondary network forms via photopolymerization among the vinyl groups on maleic-modified chitosan, polyethylene glycol diacrylate (PEGDA), and dopamine methacrylamide (DMA) to enhance mechanical properties and provide wet adhesion through the catechol moieties [66]. The resulting hydrogel maintains a high adhesive strength (35 kPa) and an integral gel state in extreme pH environments (pH = 1) for more than 7 days, owing to its strong secondary covalent network [66]. In another study, an acid-tolerant hydrogel bioadhesive integrates two distinct components: poly(2-hydroxyethyl methacrylate-co-*N*-vinylpyrrolidone) (poly(HEMA-NVP)) and poly(acrylic acid-co-*N*-hydroxysuccinimide acrylate ester) (poly(AA-NHS)) (Figure 4C) [53]. Poly(HEMA-NVP) contributes to the acid tolerance of the hydrogel through phase segregation facilitated by hydrophobic association, intrinsic van der Waals interactions, and hydrogen bonds. Poly(AA-NHS) functions as an adhesive brush to form robust and seamless interfacial adhesion through the dry-crosslinking mechanism. The three-layered structure effectively seals 5 mm perforation in a few seconds and remains stable for more than 14 days [53].

### 3.6. Hydrogel in Different Forms

In addition to the formulation and structure of the hydrogel, it is also critical to consider the shape of the hydrogel as it determines the route of administration and the efficacy of perforation sealing. Microparticle-based hydrogels are easily sprayable and endoscopically deliverable but may be inappropriate for larger perforation sizes due to their lowering of burst strength [28]. Glue-type hydrogels are suitable for minimally invasive surgeries via the endoscope or laparoscope. However, the hydrogel solution must show good injectability, quick gelation, and ease of operation. A multi-step gelation process that involves light administration and extra reactants may require specially designed delivery devices [54,67]. Patch-type hydrogels are designed for perforation sealing during open surgery and should stick instantly upon application. A major clinical problem of conventional adhesives is the undesired postoperative tissue adhesion due to indiscriminate adhesion, which causes severe consequences including chronic pain, ileus, and infertility [69]. To overcome this challenge, Janus hydrogels are being investigated with single-sided wet adhesion capabilities. A negatively charged carboxyl-containing hydrogel can be gradiently complexed with a cationic oligosaccharide via the one-sided dipping method to form a Janus hydrogel with excellent asymmetric adhesion and non-adhesion on two surfaces [69]. Alternatively, single-sided patterning with Fe^3+^ through a paper-based transfer printing method also affords Janus hydrogel patches [58]. Full blocking of the perforation defect helps the material anchor to the wound site completely and prevents adhesive failure. Liu et al. report a mushroom-cap inspired hyperboloid-shaped bioadhesive consisting of a dimethylacrylamide network crosslinked with *N*,*N*-cystaminebis(acrylamide) and a sodium alginate network crosslinked with calcium ions. The resulting hydrogel, casted into thin sheets and rolled to build a multilayer hyperboloid cap-stick-shaped device with an onion-like structure, facilitates endoscopic delivery and self-expandable full layer blocking (Figure 4D) [52].

### 3.7. Wound Healing

Almost all published hydrogel designs show good biocompatibility, complete sealing of the perforation, and superior wound healing effects compared to conventional sutures or fibrin glue in in vivo gastric perforation models, ranging from mice and rats to rabbits and pigs. The application of the adhesive hydrogel alone accelerates the transition from inflammation to proliferation, suppresses excessive fibrosis to promote ECM remodeling, and provides nutrition for angiogenesis and re-epithelization [39,53,57,59,60,62,67]. Additionally, small molecular drugs and growth factors are incorporated into the hydrogel for sustained release [52,57]. Fibroblast growth factors (FGFs) stimulate endothelial cell proliferation and angiogenesis in pathological conditions [70]. Both acidic FGF (FGF-1) and basic FGF (FGF-2), incorporated in hydrogels, promote further cell proliferation and migration, thereby accelerating abdominal wall repair (Figure 4D) [38,52]. Vonoprazan fumarate (VF), a potassium-competitive acid blocker, loaded in hydrogels, regulates intragastric pH to promote the healing of lesions [52]. Gastric acid interferes with platelet activation and deteriorates blood clots. VF inhibits the exchange of H^+^ and K^+^ in the gastric mucosa, leading to a higher local pH, a decreased risk of bleeding, and better mucosa healing [52].

### 3.8. Summary

An ideal hydrogel adhesive for GI perforation needs to exhibit high underwater adhesive strength to effectively seal the perforation, strong mechanical properties to prevent adhesive and cohesive failure, long-term extreme pH tolerance to remain stable in the GI environment, biocompatibility to ensure safety, and usability for ease of application. To ensure strong wet adhesion, researchers maximize the physical, chemical, or topological interactions between the hydrogel and the tissue surface through various strategies including: incorporation of a large numbers of physical or chemical anchoring moieties into the hydrogel network; elimination of interfacial water via the use of hydrophobic residues and dry hydrophilic scaffolds; and implementation of multiple adhesive mechanisms in parallel. To realize acid tolerance, supramolecular assemblies through hydrophobic interactions, nonhydrolyzable double covalent networks, and pH-responsive acid-resisting drugs are being explored for novel hydrogel designs. To achieve enhanced toughness, strength, and ductility, biomimetic TA-based hydrogels and IPN hydrogels with both covalent and physical crosslinking networks provide both cohesion and adhesion. Additionally, hydrogels in different forms are being investigated to accommodate various surgical delivery routes and perforation sizes. Compared to conventional sutures and bioadhesive glues, hydrogel adhesives generally show high perforation sealing efficacies and superior wound healing effects in in vivo animal models. Many formulations show promise for clinical translation. However, due to the complexity of the disease, a universal hydrogel adhesive solution for GI perforation treatment seems unlikely. Additionally, one should be cognizant of industrial scalability and the companion device needed for delivery and not create an overly complex hydrogel adhesive. Significant opportunities exist to advance better hydrogel designs and treatment outcomes for GI perforation.

## 4. Anastomotic Leak

### 4.1. Disease Condition and Current Standards

GI anastomosis is a surgical procedure performed to restore continuity between two formally distant portions of the GI tract, usually after the removal of a diseased tissue [6]. One of the deadliest complications of anastomosis is anastomotic leakage (AL), the leak of luminal contents into the abdominal cavity. The overall incidence rate varies from 6% to 20% with complications such as sepsis, peritonitis, or multiple organ failure resulting in a mortality rate between 13% and 27% [18,71,72]. AL etiology is multifactorial, mostly based on ischemia of the bowel endings and/or technical failure [73]. Risk factors for AL include patient-related factors, such as comorbidity, high body mass index, and nonsteroidal anti-inflammatory drug (NSAID) use, as well as intraoperative factors such as surgical technique, anastomotic location, and operation time [71,73]. Currently, the most common methods for detecting AL are radiological techniques such as computerized tomography scans and water-soluble contrast enema [71,74].

The severity of the leak and the clinical condition of the patient govern the management of AL, ranging from nonoperative to operative interventions [75]. Nonoperative managements include parenteral nutrition, broad-spectrum antibiotics, and radiological drainage [72]. Traditional operative intervention for AL is the resection of the anastomosis with exteriorization of the proximal limb as an end colostomy, also known as the Hartmann’s procedure [75]. Recently, laparoscopic anastomotic surgery is gaining attention as an alternative to open surgery due to the many advantages of minimally invasive surgery [71]. Endoscopic techniques are relatively new conservative options that reduce the necessity for a stoma or can be performed as supplementary-to-surgery treatments to enhance the healing process [72]. The most commonly applied endoscopic solutions are endoscopic clipping, suturing, stenting, and vacuum-assisted endosponge therapy [72,75]. Despite their high success rate (about 80–90%), they are limited to small leaks, require additional anchoring reinforcements, and can cause bleeding, perforation, and adjacent injuries [72]. In clinical research, fibrin glue, cyanoacrylate, PEG-based hydrogel, and other commercially available adhesives have been used to reinforce the stapling and suturing lines to seal GI anastomosis [73]. However, the containment of the leaks by such materials is inadequate due to their susceptibility to degradation in GI fluid (e.g., fibrin and gelatin) and the mechanical mismatch with the tissue (e.g., cyanoacrylate, PEG hydrogels) [6,10,20,76]. Additionally, current sealing agents exhibit low wound healing capacity and may pose risks of cytotoxicity, as a result, failing to reduce the incidence of complications after GI anastomosis [6,20]. Therefore, more advanced tissue adhesives need to be developed to either support the conventional suturing-and-stapling procedure or seal the leakage in a sutureless manner while assisting in long-term wound healing.

### 4.2. Hydrogel Requirements

Hydrogel adhesives reinforce the suturing and stapling lines to prevent AL by mechanically supporting the anastomotic site, containing the leak, or promoting anastomotic healing. The design requirements for such hydrogel adhesives include: (1) strong mechanical strength; (2) good adhesive performance; (3) bioactive scaffold to promote wound healing; (4) anti-adhesive backing layer; (5) ease of application and use; and (6) biocompatibility. Wound healing in the GI tract is different from that of cutaneous healing and is not yet fully understood [71]. The highest risk for AL lies in the first 2 to 4 days post-surgery, during which fibroblast infiltration and collagen deposition start to take place [71]. Therefore, it is beneficial to increase the mechanical strength of the anastomosis (burst pressure > intra-abdominal pressure 25 mmHg in vivo) to resist the fluid pressure caused by bowel movements during this period before anastomosis becomes sufficiently stabilized [59,77]. Bioactive and porous three-dimensional hydrogel constructs enhance regeneration of the sutured site by providing a mechanically stable and hydrophilic environment conducive to cell attachment, proliferation, and differentiation [78]. An additional hydrogel design is the introduction of an anti-adhesive backing layer to prevent post-operative peritoneal adhesion, which causes abdominal discomfort or even more serious complications such as ileus, necessitating reoperation [79].

Although suturing and stapling allow for complex and life-saving reconnections, its inherent disadvantages such as technical complexity and risks of unwanted side effects prompt researchers to develop atraumatic sutureless hydrogel adhesives for GI anastomosis [20]. However, sutureless GI anastomosis is exceptionally challenging due to the instability of anastomosed tissue and the complexity of the surgical condition, including the difficulty involved with different orientations of the bowel [20,71]. An ideal hydrogel adhesive for sutureless GI anastomosis requires: (1) strong and stable long-term wet adhesion; (2) tolerance for chemically harsh digestive conditions; (3) mechanically robust, moderately swelling network; (4) excellent interfacial toughness, ductility, and fatigue resistance; (5) anti-adhesive backing layer; (6) optimal dimensions to facilitate ease of application; and (7) biocompatibility. The design requirements here are even more demanding than those for the repair of GI perforations because the hydrogel adhesive will need to withstand the circumferential fluid pressure and resist the corrosion of digestive fluids on its own without the support of any GI tissue. The strength of the anastomotic site depends on the presence of collagenous networks produced by fibroblasts and usually only starts to match those of the healthy tissues after 7 days [71,77]. Therefore, the hydrogel adhesive should remain adhered to the disjointed tissues and endure the chemically harsh and mechanically fluctuating GI environment for at least 7 days. In addition to the stringent mechanical properties needed for prolonged adhesion, including strong wet adhesion (adhesive strength > 10 kPa), robust cohesive networks (G′ > 6 kPa), and good energy dissipation (interfacial toughness > 50 J/m^2^), hydrogel adhesives designed for sutureless anastomosis need to have high burst pressure to inhibit fluid leakage (burst pressure > 25 mmHg in vivo), controlled swelling to avoid the mismatch strain between the hydrogel and the tissue, and a nonadhesive backing layer to prevent peritoneal adhesion [10,80]. Excessive swelling of the hydrogel (likely > 50 wt%) will cause weakening of the hydrogel network and separation of approximated wound edges, causing subsequent leakage or delayed healing [10]. Only hydrogel adhesives that meet all the aforementioned standards may potentially secure GI anastomotic leaks in a sutureless manner.

### 4.3. Hydrogels for Suture/Staple Reinforcement

Three-dimensional (3D) polymeric scaffolds enhance wound healing by creating an appropriate microenvironment similar to the ECM for native cells to proliferate, migrate, and differentiate [81,82]. These scaffolds comprise synthetic and natural polymers such as poly(lactic-co-glycolic) acid (PLGA), PCL, PEG, PVA, collagen, chitosan, fibrin, and gelatin [83]. The combination of synthetic and natural polymers is of special interest for wound healing applications due to the mechanical strength and tunable degradability of synthetic polymers and the bioactivity and biocompatibility of natural polymers [84]. Recently, the application of 3D polymeric scaffolds to reinforce suturing and stapling lines during surgical anastomosis has started being investigated. Fabrication techniques for these scaffolds include freeze-thawing, blow-spinning, dry spraying, and electrospinning [76,78,79,85,86].

Electrospinning affords PCL-gelatin and PVA-PCL nanofibrous patches to cover anastomotic sutures and improve healing [78,79]. While both studies show successful leakage prevention and no complications in in vivo anastomosis models, the results in wound healing are mixed as greater inflammation and less re-epithelialization occur with the PVA-PCL patch compared to the control [79]. The volatile acid solvents used during electrospinning compromise cell viability, if not completely removed [78]. To induce a more porous surface for enhanced tissue bonding, Dorkhani et al. describe PVA films with patterned gelatin particles [85]. The sealant film shows excellent mechanical properties and active regulation of cytokines, such as lower levels of tumor necrosis factor α (TNF-α), and higher levels of transforming growth factor β (TGF-β), resulting in more wound healing and collagen deposition [85]. Blow spinning is an easy-to-perform technique that allows the deposition of polymer fibers to conform to specific anatomical geometries, thus enhancing surgical usability [76]. A blow-spun polymer composed of PLGA and PEG shows high adhesive strength (120 kPa) and high burst pressure (37 mmHg) in a piglet sutured intestinal anastomosis model owing to the softening fiber mat to film transition that increases polymer-substrate interactions at body temperature [76]. In addition to suture reinforcement, Paonessa et al. report ring-shaped hydrogel structures to support entero-entero (EEA) circular stapling after swelling [86]. Ring-shaped PVA-gelatin hydrogels, prepared through a physical freeze-thawing crosslinking method which eliminates the addition of toxic agents, ensure better compliance with the bowel tissue [86]. The limitations of conventional polymeric scaffolds, however, lie in their inability to adhere to completely wet surfaces and therefore the possibility of obstructing the bowel [76].

**Figure 5:**
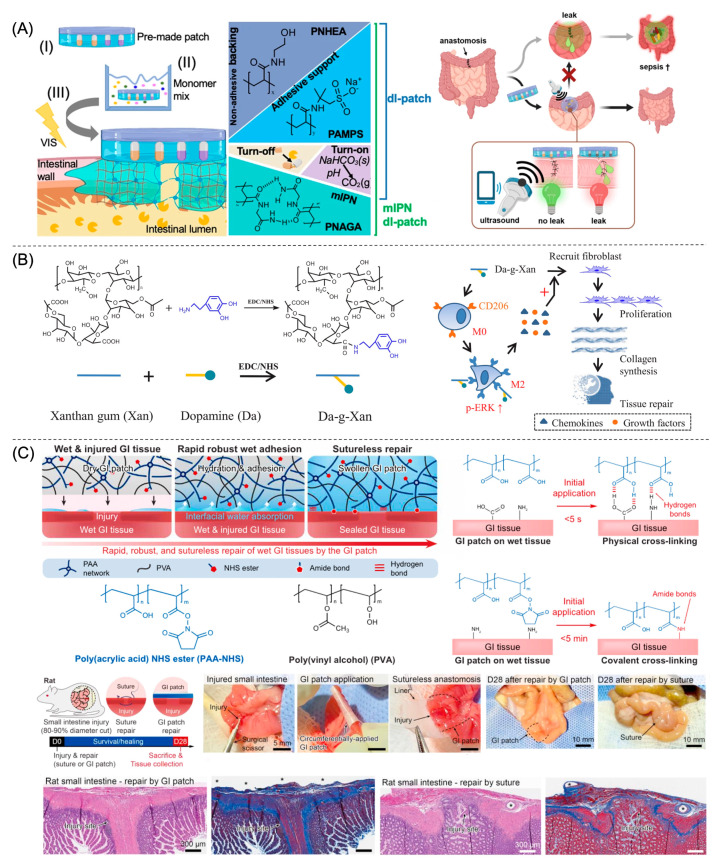
Hydrogel adhesives designed to treat anastomotic leaks. (**A**) A modular, intelligent suture support sealant patch is capable of tissue adhesion through its mIPN and leak detection through its ultrasound sensing elements, reprinted with permission from Ref. [88]. Copyright 2022 Springer Nature. (**B**) A marine-inspired hydrogel adhesive based on the underwater adhesive mechanisms of mussels and barnacles augments native tissue regenerative responses by inducing M2 polarization, reprinted with permission from Ref. [87]. Copyright 2021 Elsevier. (**C**) An off-the-shelf bioadhesive GI patch capable of atraumatic, rapid, robust, and sutureless repair of GI defects shows its efficacy in a rat small intestine anastomosis model, reprinted with permission from Ref. [10]. Copyright 2022 American Association for the Advancement of Science.

Hydrogels with wet tissue adhesion capabilities are ideal as suture reinforcement materials since they can assist in containing potential anastomotic leaks. Huang et al. report a biomimetic hydrogel adhesive composed of dopamine-conjugated xanthan gum to synergistically mimic the underwater adhesive mechanisms of mussels and barnacles (Figure 5B) [87]. The hydrogel exhibits good injectability, self-healing properties, and strong underwater adhesive strength (27 kPa) due to its abundant intermolecular hydrogen bonding interactions and the formation of multiple interfacial linkages, including hydrogen bonds, carbon-sulfur bonds, Michael addition, amide bonds, and imine bonds [87]. These different bonding motifs contribute to its high burst pressure (120 mmHg) and stability in reinforcing in vivo anastomotic sutures in rats [87]. Alternatively, Anthis et al. ensure leak containment and resistance to harsh digestive conditions by implementing a mutually interpenetrating network (mIPN) traversing a pre-made hydrogel patch and the tissue simultaneously to firmly anchor the patch onto the tissue (Figure 5A) [88]. The pre-made hydrogel consisting of adhesive polyanionic polymer poly(2-acrylamido-2-methyl-1-propanesulfonic acid) sodium salt (PAMPS) is soaked in a precursor solution of *N*-acryloyl glycinamide (NAGA) and its photoinitiator lithium phenyl-2,4,6-trimethylbenzoylphosphinate (LAP). Upon patch application and light irradiation, a robust mIPN of the non-adhesive polymer PNAGA forms to join the pre-made hydrogel with the tissue [88]. As a result, the hydrogel achieves high adhesion energies and high burst pressures against porcine small intestine, stomach, and colon, as well as remains mechanically robust after incubation with digestive fluid for 24 h [88].

### 4.4. Wound Healing

Similar to general wounds, anastomotic healing occurs in three overlapping phases: (1) hemostasis and inflammation; (2) proliferation or granulation; and (3) remodeling or maturation, but in a slightly faster fashion [83,89]. The hemostasis and inflammation phase occurs in the first 4 to 5 days characterized by the stabilization of the fibrin clot and the infiltration of inflammatory cells [89]. The following proliferative phase continues for about two weeks to regenerate granulation tissue through collagen deposition, fibroblast proliferation, and neovascularization [82,89]. The GI wall layer completely reorganizes and stabilizes in the final remodeling phase, usually two weeks post-surgery [89].

To enhance anastomotic healing in all three stages, researchers incorporate a variety of growth factors and drugs into the hydrogel. Hydrogel formulations loaded with anti-infective, anti-microbial, and anti-inflammatory drugs such as gentamycin, polyvinyl pyrrolidone-iodine (PVP-I), and acetylsalicylic acid accelerate the transition from inflammation to proliferation [86,88,90]. However, contradictory evidence exists concerning the negative effects of NSAID drugs on GI wound healing, restricting their regulatory uses [71,91]. Macrophages play a central role in the first two stages of wound healing, regulating inflammation by altering cellular polarization from type 1 macrophage (M1) to type 2 macrophage (M2). The free dopamine-conjugated xanthan gum degraded from the hydrogel, designed by Huang et al., regulates the inflammatory status and induces type 2 macrophage polarization (M2) by binding with endocytic mannose receptor CD206 on the macrophage and increasing the downstream extracellular regulated protein kinase (ERK) signaling (Figure 5B) [87]. Consequently, M2 macrophages secrete larger amounts of chemokines and growth factors, strengthening fibroblast migration and proliferation, collagen synthesis, and epithelial vascularization, thereby protecting surgical anastomosis [87]. Alternatively, hydrogels release growth factors such as FGFs to enhance the proliferation stage [77]. Bioactive silicon ions also promote angiogenesis and enhance GI tissue regeneration [92].

### 4.5. Anti-Adhesion

Postoperative peritoneal adhesion (PA) is a serious complication following general abdominal surgeries, occurring in more than 90% of patients and causing bowel obstruction, infertility, chronic pain, and difficult re-operative surgeries [93]. During anastomotic healing, the development of coagulation, inflammation, and fibrinolysis can induce over-deposition of fibrin which results in the formation of PA [94]. The application of hydrogels as physical barriers to separate affected areas in the early postoperative period is of significant clinical utility [15]. Therefore, in addition to the adhesive layer that attaches to the suture/staple line to reinforce sealing and promote healing, a non-adhesive backing layer is necessary to prevent excessive swelling and PA. Hydrophilic polyurethane, PEG, PCL, and poly(*N*-hydroxylethyl acrylamide) (PNHEA), among others, are common non-adhesive backing layer materials due to their biocompatibility and anti-fouling properties which prevent protein attachment [10,79,88,95]. Additionally, in situ forming hydrogels, based on mechanical interlocking adhesion mechanisms, exhibit asymmetric adhesive and non-adhesive surfaces because the cured flat hydrogel will have less contact with surrounding rough tissues [80]. The design for both suture reinforcement materials and sutureless hydrogel patches should consider possible nonspecific adhesion between the anastomotic site and the surrounding tissues.

### 4.6. Sutureless Anastomosis

Complete sutureless GI anastomosis via hydrogel adhesives is extraordinarily difficult because the sealant is directly exposed to complex digestive contents and experiences the volatile mechanical motions of GI motility without any tissue support. To date, there is no research demonstrating the success of hydrogel adhesives to solely anastomose two disjointed sections of the GI tract. However, many hydrogel adhesives seal anastomotic leaks suturelessly through their enhanced mechanical properties, excellent wet adhesion mechanisms, and anti-degradation capabilities. For example, Alaska pollock-derived gelatin modified with decyl groups (C10) and crosslinked with tetra-PEG succinimidyl glutarate polymerizes in situ within 10 s and adheres to tissue strongly through chemical bonding and hydrophobic interactions [80]. The hydrophobic modification suppresses the swelling ratio and enhances the burst pressure (52.5 mmHg) [80]. Anthis et al. report a chemically resistive, leak-tight, and mucoadhesive hydrogel sealant that harnesses the synergistic effect between mucoadhesion and mechanical fixation to anchor the gels to the anastomotic site [18]. The hydrogel components, copolymerized acrylamide, acrylic acid, methyl acrylate, and bis-acrylamide, ensure mucoadhesive character, hydrophobic nature, and stability towards degradation, respectively [18]. Brief incubation of the prepared poly(acrylamidemethyl acrylate-acrylic acid) (pAAm-MA-AA) hydrogel in its precursor monomer solution, followed by application to the intestinal tissue and light irradiation, affords an interpenetrating network that traverses both the hydrogel and the tissue, and effectively seals an ex vivo anastomotic leak in simulated intestinal fluid (pH 6.8) for more than 24 h, compared to 5 min for commercially available fibrin glue [18]. Unfortunately, in vivo experiments are lacking in the previous two studies to validate the efficacy of their hydrogel adhesives, partially because currently available rodent models of intestinal anastomotic leakage are poorly established and suffer from high variability [18,96].

Currently, there is no single animal model appropriate for bowel anastomosis with regards to practical ease, costs, reproducibility, and clinical translation [96]. Despite the insufficient knowledge about GI anastomosis, experts in the field reach consensus on several recommendations, where mouse, rat, and pig models of colon, stomach, and small intestine anastomosis usually manifested as linear incisions (<10 mm) or circular defects (<5 mm in diameter) are considered appropriate [10,95,96]. Jeon et al. describe a hydrogel-forming double-layered adhesive microneedle patch (MN) consisting of a swellable mussel adhesive protein (MAP) and hyaluronic acid coacervate shell and a non-swellable silk fibroin core crosslinked with interfacial dityrosine [95]. Surface microtopography and the intrinsic strong adhesion of MAP act synergistically to exert substantial adhesion onto wet and dynamic biological surfaces via swelling-mediated mechanical interlocking and diverse physical and chemical interactions [95]. In a rat ileum defect model of 5 mm diameter, the MN successfully seals the leakage, maintains adherence for more than 7 days, and accelerates submucosa regeneration with angiogenesis and smaller lymphoid follicles, while the sutured group shows massive granulation tissues and prolonged inflammation, suggesting the excellent wound healing capability of the MN [95]. 

In another study, Wu et al. report a hydrogel patch consisting of a nonadhesive top layer of hydrophilic polyurethane and a bioadhesive layer of IPNs between covalently crosslinked PAA-NHS for dry adhesion and physically crosslinked PVA for mechanical reinforcement (Figure 5C) [10]. Patch application provides instant, atraumatic, and fluid-tight sealing of 10 mm incisional defects in rat colon and stomach, lower degrees of fibrosis and inflammatory response, and elevated levels of collagen deposition [10]. Markedly, in a rat small intestine anastomosis model where approximately 90% of the diameter is cut, the circumferentially applied hydrogel patch also forms complete sutureless sealing and assists in wound healing [10]. Considering the resemblance of this animal model and actual anastomosis, where the GI tract is completely disjointed, the positive results of this study demonstrate the substantial potential of hydrogel adhesives for future sutureless GI anastomosis.

### 4.7. Leak Detection

One typically investigates anastomotic leaks using radiological imaging modalities such as computerized tomography and contrast radiography or through the assessment of manifested clinical symptoms such as tachycardia and hyperthermia, which introduce delays before AL treatment commences [88,97]. Continuous postoperative monitoring of the anastomotic site is thus necessary for early and unambiguous identification of AL. Hydrogels can facilitate anastomotic leak detection by either protecting or delivering the sensing element [88,97]. Insufficient tissue oxygenation, indicative of tissue ischemia, is a predictive sign of GI AL [97]. Marland et al. describe a miniature implantable electrochemical oxygen sensor consisting of an array of platinum microelectrodes microfabricated on a silicon substrate and a poly(2-hydroxyethyl methacrylate) hydrogel membrane that continuously monitors tissue oxygenation in a pig colorectal anastomosis model for over 45 h [97]. The hydrogel coating serves as a biocompatible element that mitigates the effects of biofouling and facilitates oxygen diffusion into the sensor [97]. Anthis et al. describe a GI leak sensing hydrogel sealant capable of identifying the digestive fluid breaching of sutures as early as 3 h ex vivo (Figure 4A) [88]. The hydrogel contains two conceptually distinct embedded ultrasound sensing elements: (1) enzymatically digestible gas-filled proteinaceous structures in a soft acrylamide matrix for enzyme-responsive TurnOFF sensing; and (2) agar matrix dissolved acid-reactive sodium bicarbonate for pH-responsive TurnON sensing [88]. Unfortunately, the ultrasound signals are not discernible in vivo and thus require further design adjustments [88].

### 4.8. Summary

Due to the substantial risk of GI AL, hydrogel adhesives reinforce the conventional suturing-and-stapling procedure by mechanically fortifying the anastomotic site and promoting wound healing. Porous and fibrous polymeric scaffolds, fabricated from a variety of natural and synthetic polymers, increase the strength of the anastomotic site and facilitate cell infiltration. Biomimetic adhesive moieties and mechanical interlocking mechanisms enhance wet adhesion. To further accelerate wound healing, growth factors and drugs are incorporated into the hydrogel. Additionally, hydrogels reduce postoperative peritoneal adhesion and facilitate AL detection. Although challenging, sutureless sealing of AL is possible with biocompatible, biodegradable, and highly adhesive hydrogel patches but requires further refinement [10,97]. The research for hydrogels for AL prevention and GI anastomosis is still in its infancy. Knowledge regarding the pathophysiology of AL and the anastomotic healing process is lacking and reliable anastomotic animal models are not fully established [96,98]. The results of recent studies, however, demonstrate significant promise for hydrogels to serve as a potential solution for AL prevention with or without sutures.

## 5. Concluding Remarks

GI diseases are a major cause of morbidity and mortality worldwide, affecting 60 to 70 million people in the United States alone and hundreds of millions worldwide [99]. Approximately 30% of the affected population go through some type of GI surgery to remove the diseased tissue and to restore the health and continuity of the digestive system [99]. Therefore, effective and atraumatic closure and repair of GI wounds is of significance in reducing post-operative complications and improving the quality of life for patients. 

In the past few years, there has been marked progress in the development of hydrogel adhesives for treating different types of GI wounds. To treat acute GI bleeding, hydrogel designs emphasize injectability, quick gelation, infection prevention, and hemostatic effects. Many in situ gelation mechanisms enable rapid crosslinking and easy endoscopic delivery. To seal GI perforations, researchers focus on enhancing the hydrogel’s mechanical properties and wet adhesion capabilities to prevent adhesive and cohesive failures in the GI environment. Diverse strategies to simultaneously maximize the physical, chemical, and bionic interactions between the hydrogel and the tissue as well as the strength, extensibility, and fatigue resistance of the device include hydrophobic modifications, supramolecular assemblies, and IPN hydrogel designs. To prevent anastomotic leaks, suture reinforcement hydrogel adhesives exhibit greater burst strength and utilize bioactive polymer scaffolds incorporated with drugs or growth factors to accelerate wound healing. On the other hand, sutureless hydrogel adhesives underscore the importance of prolonged wet adhesion in chemically harsh conditions by using slow-degrading polymers with strong adhesive properties.

Despite the advancements and successes, ample opportunities remain for further improvements. Achieving GI tissue adhesion for over 48 h in vivo is extremely difficult, predominantly owing to the continuous mechanical motions of the GI tract and the chemically harsh digestive environment, where hydrogen bonding and pH-sensitive mechanisms may not be sufficient. In most cases, long-lasting strong wet adhesion is achieved by applying a patch during open surgery or using hydrogels that degrade slowly. However, these methods may result in foreign body response and mild levels of inflammation and may not be feasible for minimally invasive procedures. For hydrogels intended for endoscopic or laparoscopic delivery, their complicated gelation steps add extra technical difficulties and increase the duration of the surgical procedure, rendering them unfavorable to treat large wounds. More generally, the intricate designs of multi-functional hydrogel adhesives presented in this review pose challenges for their mass production and practical implementation in clinical settings. Additionally, complete sutureless anastomosis using hydrogel adhesives remains impossible. Elucidating the biological processes behind GI wound healing will better inform the design of hydrogel adhesives to seal GI defects and promote GI wound healing. Nonetheless, as discussed in this review, many hydrogel adhesives demonstrate efficacy in GI wound closure and repair in animal models. Future research that addresses the aforementioned limitations will advance hydrogel adhesives to the clinic for GI wound care with the goal of establishing a new standard of care for millions of patients.
